# Polymorphisms of the renin-angiotensin system are not associated with overweight and obesity in a general adult population

**DOI:** 10.20945/2359-3997000000155

**Published:** 2019-07-11

**Authors:** Deborah de Farias Lelis, Alexandre Costa Pereira, José Eduardo Krieger, José Geraldo Mill, Sérgio Henrique Sousa Santos, Marcelo Perim Baldo

**Affiliations:** 1 Universidade Estadual de Montes Claros Programa de Pós-Graduação em Ciências da Saúde Universidade Estadual de Montes Claros Montes Claros MG Brasil Programa de Pós-Graduação em Ciências da Saúde, Universidade Estadual de Montes Claros, Montes Claros, MG, Brasil; 2 Universidade de São Paulo Instituto do Coração Universidade de São Paulo São Paulo SP Brasil Instituto do Coração, Universidade de São Paulo, São Paulo, SP, Brasil; 3 Universidade Federal do Espírito Santo Departamento de Ciências Fisiológicas Universidade Federal do Espírito Santo Vitória ES Brasil Departamento de Ciências Fisiológicas, Universidade Federal do Espírito Santo, Vitória, ES, Brasil; 4 Universidade Federal de Minas Gerais Instituto de Ciências Agrárias (ICA) Universidade Federal de Minas Gerais Belo Horizonte MG Brasil Instituto de Ciências Agrárias (ICA), Engenharia de Alimentos, Universidade Federal de Minas Gerais, Belo Horizonte, MG, Brasil; 5 Faculdades Integradas Pitágoras Departamento de Medicina Faculdades Integradas Pitágoras Montes Claros MG Brasil Departamento de Medicina, Faculdades Integradas Pitágoras, Montes Claros, MG, Brasil

**Keywords:** Renin-angiotensin system, obesity, polymorphism

## Abstract

**Objective:**

The increased prevalence of obesity and associated comorbidities, such as cardiovascular and metabolic diseases, has gained attention worldwide, and the renin-angiotensin system (RAS) has been pointed out as a possible link. Thus, the present study aimed to verify the possible association between angiotensinogen (AGT) or angiotensin-converting enzyme (ACE) polymorphisms with overweight and obesity in adults.

**Subjects and methods:**

The present investigation was a population-based cross-sectional study including 1,567 individuals from an urban area in Brazil. Anthropometric, clinical and biochemical parameters were evaluated, and all individuals were genotyped for the ACE I/D and AGT M/T polymorphisms.

**Results:**

The prevalence of overweight was higher among men, whereas obesity was more prevalent among women. However, the frequency of ACE or AGT polymorphisms was similar among body mass index (BMI) categories. In addition, the mean age-adjusted BMI averages did not change significantly for ACE or AGT polymorphisms, regardless of sex or BMI category. The age-adjusted BMI average for the combination of ACE and AGT genotypes evidenced no significant differences regardless of sex or BMI categories. Results were similar when BMI was replaced by waist circumference (WC).

**Conclusions:**

We were not able to find any associations between BMI and WC (overweight/obesity) and ACE and AGT polymorphisms, indicating that the RAS system might not be involved in overweight and obesity, at least based on genetic backgrounds. However, further studies must measure RAS components to elucidate this question.

## INTRODUCTION

The prevalence of overweight, obesity and related cardiometabolic diseases is increasing worldwide and has been reported as a modern pandemic (1). According to recent data from the World Health Organization (WHO), obesity has almost tripled since 1975, and in 2016, approximately 2 billion adults were overweight/obese worldwide (2). Obesity is characterized by excessive fat accumulation that leads to health complications and is strongly associated with development of cardiovascular diseases (CVDs) (2,3). Risk factors for CVDs such as insulin resistance, hypertension, inflammation and high plasma lipid levels are associated with increased fat mass accumulation and obesity. Moreover, obesity induces several alterations in cardiac structure and function, predisposing individuals to develop cardiovascular complications such as heart failure, coronary heart disease and myocardial infarction, among others (3).

According to some authors, the renin-angiotensin system (RAS) constitutes a possible link between obesity and CVDs (4-6). Indeed, the increased expression of some RAS components has been associated with CVDs and obesity (7). The gene expression of RAS components is increased in the visceral adipose tissue of obese humans (8); the production of angiotensin II (AngII) also increases with obesity (7). This peptide stimulates the proliferation of the vascular smooth muscle and induces cardiomyocyte hypertrophy, endothelium dysfunction and vasoconstriction, which all lead to cardiac and vascular remodeling (9). Accordingly, the increased activity of the angiotensin-converting enzyme (ACE) is linked to increased AngII levels that represent the majority of the deleterious effects of the ACE/AngII/AT1 RAS axis (10).

Blood levels of RAS components are mainly regulated by water and sodium balance but are also affected by genetic variants. Genetic polymorphisms of RAS components have been associated with several cardiovascular and metabolic conditions, such as hypertension and metabolic syndrome (11,12). However, although some authors have addressed this issue in past years, there is no consensus on the effects of some RAS polymorphisms on overweight and obesity. For instance, Thomas and cols. (12) showed that the D-allele of the ACE insertion/deletion polymorphism was correlated with hypertension and obesity in boys but not girls. Moreover, the ACE II homozygosis was reported as predictor of extreme obesity and diabetes (13). Regarding the angiotensinogen (AGT) gene, the T/T genotype of the AGT M235T gene polymorphisms positively associated to visceral obesity and insulin levels in obese Japanese women (14). However, some other studies have not found the same association (15-17). These varying results might be due to different study designs, sampling methods and specific populations. From this perspective, the present study aimed to verify whether the AGT or ACE polymorphisms were associated with overweight and obesity in a population-based study in Brazil.

## SUBJECTS AND METHODS

### Study population

The present investigation was a population-based cross-sectional study (MONICA/Vitória) including adult individuals (25 to 64 years old) living in Vitória, Espírito Santo, Brazil. The study sampling and design details are described elsewhere (18,19). Briefly, the sample plan was established following a multistage probability sampling, taking into account the seven administrative regions of the city of Vitória-ES. The number of individuals allocated within each stratum is proportional to the number of residents as estimated by the city hall. The seven administrative regions include 79 neighborhoods; in each neighborhood, the census sections of IBGE were identified and drawn. A randomization mechanism was used to identify the households within each census section to provide individuals for the final sample. Individuals were invited to participate in the study and were included after signing the free informed consent. The sampling plan aimed to include individuals from all socioeconomic classes and both sexes.

A total of 1,662 individuals attended clinical and laboratorial assessments at the university hospital. This study was submitted to and approved by the institutional ethics committee, and all participants signed a free informed consent.

### Clinical and biochemical assessment

The anthropometric parameters were assessed as previously detailed (20). The waist circumference (WC) was measured at the midpoint between the last costal arch and the iliac crest and the maximum point of expiration, while the hip circumference was measured around the thighs at the greater trochanter height; both were measured while individuals were standing. A wall stadiometer (0.5 cm accuracy) was used for height measurement and a calibrated scale (0.1 kg) was used for body weight measurement. The ratio of body weight (kg) to height squared (m^2^) was used for BMI calculation.

Heart rates were measured by counting pulses for 30 seconds. Blood pressure was assessed with a mercury sphygmomanometer in the left arm in fasting individuals in a sitting position after 5 to 10 minutes resting. Systolic (SBP) and diastolic (DBP) blood pressure were measured in the first and fifth Korotkoff phases, respectively. Three readings were obtained from each participant at 2-3 min intervals, and the clinic blood pressure was calculated as the mean of the two last readings.

The biochemical blood parameters were assessed via venipuncture in an upper limb following a 10- to 14-hour fasting period. The analyses were performed with commercially available kits in the central laboratory (LabSESI-Vitória). Glucose (tube containing fluoride) and lipid (ethylenediamine tetra-acetic acid (EDTA) preparations were used for various biochemical parameters. The Friedewald equation was used to estimate LDL-c lipoprotein when triglycerides were < 400 mg/dL.

### Genotyping

Genomic DNA was extracted from leukocytes in samples of whole blood, following a standard salting-out procedure (21). The ACE gene I/D polymorphism was determined through a 3-primer system and the M235T variant of the AGT gene by a standard PCR detection method. The ACE gene I/D polymorphism is based on the insertion (I) or deletion (D) of a 287-bp sequence of DNA (in the ACE gene-intron 16) (NCBI ref. SNP ID: rs1799752), while the AGT M235T variant is a polymorphism (NCBI ref SNP ID: rs699) (T/C at position +704) that converts methionine into threonine at amino acid 235. Quality control for these assays was assessed by randomly selecting 50 samples to be re-genotyped by two independent technicians. No misgenotyping was observed in this sample.

### Statistical analysis

The statistical package SPSS (version 22.0, Chicago, IL, USA) was used to perform the statistical analysis. Continuous variables were described as mean ± standard deviation while dichotomous variables were presented as frequency and percentage. The Kolmogorov-Smirnov test was applied to evaluate Gaussian distribution. The chi-squared test was used to compare proportions and test for Hardy-Weinberg disequilibrium. An analysis of variance (ANOVA) was followed by Tukey’s post-hoc test in the case of a significant F test. A covariance analysis (ANCOVA) was applied to test the association between BMI (and WC) with ACE and AGT polymorphism, controlling for age. The study power was calculation based on comparison between means or proportions, as reported elsewhere (22,23). The statistical significance was set as *P* < 0.05 for proportions and means.

## RESULTS

The final sample consisted of 1567 participants (women: 54%) with an average age of 44.8 ± 10.9 years. Ninety-five participants were excluded based on missing genotype data. The anthropometric, biochemical and clinical characteristics are described in Table 1. According to BMI stratification, overweight was more prevalent in men than in women (40.5% vs 32.7%, *P* < 0.033). However, obesity was found to be more prevalent in women than in men (16.1% vs 22.2%, *P* < 0.015). Moreover, men and women were similar in age, fasting blood glucose, total cholesterol and LDL-c levels.

**Table 1:** General characteristics of the sample

	MEN (715)	WOMEN (852)	P value	ALL (1567)
Age (years)	44.7 ± 10.9	44.8 ± 10.7	0.865	44.8 ± 10.9
Weight (kg)	74.0 ± 14.3	65.1 ± 15.2	< 0.001	69.2 ± 15.5
Height (cm)	169.4 ± 7.2	156.9 ± 6.2	< 0.001	162.6 ± 9.1
BMI (kg/m^2^)	25.8 ± 4.1	26.6 ± 5.5	0.003	26.3 ± 4.9
WC (cm)	88.7 ± 12.8	83.0 ± 14.6	< 0.001	85.6 ± 14.1
Waist-to-hip ratio	0.92± 0.08	0.84 ± 0.08	< 0.001	0.87 ± 0.09
Glucose (mg/dL)	105.7 ± 28.6	104.4 ± 34.5	0.400	105.0 ± 32.0
Uric acid (mg/dL)	5.48 ± 1.46	4.22 ± 1.28	< 0.001	4.80 ± 1.51
Cholesterol (mg/dL)	214.1 ± 51.2	214.7 ± 44.7	0.808	214.4 ± 47.8
LDL-c (mg/dL)	140.3 ± 40.9	142.3 ± 40.9	0.352	141.4 ± 40.9
HDL-c (mg/dL)	42.1 ± 12.2	48.0 ± 11.8	< 0.001	45.4 ± 12.3
Triglycerides (mg/dL)	160.7 ± 162.8	117.6 ± 84.0	< 0.001	137.3 ± 127.9
SBP (mmHg)	130.0 ± 19.5	126.2 ± 23.5	0.001	127.9 ± 21.9
DBP (mmHg)	86.8 ± 13.7	81.8 ± 14.4	< 0.001	84.1 ± 14.3
Overweight, n (%)	290 (40.5)	279 (32.7)	0.033	569 (36.3)
Obesity, n (%)	115 (16.1)	189 (22.2)	0.015	304 (19.4)

BMI: body mass index; WC: waist circumference; WHR: waist-to-hip ratio; LDL-c: low-density lipoprotein cholesterol; HDL-c: high-density lipoprotein cholesterol; SBP: systolic blood pressure; DBP: diastolic blood pressure; ACE: angiotensin-converting enzyme; AGT: angiotensinogen.

Table 2 displays the clinical and anthropometric characteristics stratified by sex and ACE polymorphisms (DD, DI and II genotypes). Anthropometric markers of obesity were similar among genotypes, regardless of sex. Moreover, we observed lower HDL-c levels in men with the ACE DI genotype compared to DD. When the association was tested with WC, results were similar. The remaining characteristics were similar among groups.

**Table 2:** Population characteristics according to ACE and AGT polymorphisms

ACE	MEN				WOMEN			
	
	DD (n = 213)	DI (n = 401)	II (n = 95)	P	DD (n = 244)	DI (n = 455)	II (n = 156)	P
Age	43.85 ± 11.13	45.08 ± 10.80	45.80 ± 11.27	0.265	45.73 ± 10.54	44.51 ± 11.0	44.47 ± 10.40	0.318
Weight	75.25 ± 15.39	73.49 ± 13.64	74.98 ± 14.66	0.302	65.10 ± 16.11	65.53 ± 15.65	63.85 ± 12.48	0.493
Height	169.56 ± 7.14	169.47 ± 7.13	168.95 ± 7.69	0.780	156.85 ± 6.05	157.16 ± 6.32	156.33 ± 6.25	0.355
BMI	26.21 ± 4.24	25.69 ± 3.99	26.19 ± 4.36	0.256	26.79 ± 5.57	26.68 ± 5.58	26.16 ± 5.07	0.503
WC	89.20 ± 13.34	88.48 ± 12.89	89.73 ± 11.45	0.628	82.85 ± 15.67	83.04 ± 14.79	83.26 ± 12.42	0.944
WHR	0.92 ± 0.07	0.92 ± 0.08	0.92 ± 0.07	0.937	0.84 ± 0.08	0.83 ± 0.08	0.84 ± 0.09	0.276
Glucose	107.25 ± 32.25	104.94 ± 26.70	107.18 ± 29.04	0.579	105.23 ± 38.52	103.99 ± 32.63	104.13 ± 33.98	0.899
Uric acid	5.46 ± 1.47	5.52 ± 1.43	5.52 ± 1.60	0.869	4.19 ± 1.28	4.28 ± 1.30	4.12 ± 1.26	0.383
TC	216.18 ± 63.44	213.14 ± 44.32	216.13 ± 48.97	0.740	214.26 ± 45.32	213.91 ± 44.27	218.32 ± 45.12	0.554
LDL-c	137.34 ± 39.31	141.65 ± 41.97	143.49 ± 40.67	0.374	141.12 ± 39.46	142.00 ± 42.57	145.66 ± 37.75	0.537
HDL-c	43.83 ±13.71	40.97 ± 9.97*	42.60 ± 14.78	0.020	48.43 ± 13.41	48.10 ± 11.17	47.07 ± 10.98	0.527
TG	171.62 ± 210.92	154.09 ± 99.12	170.01 ± 241.39	0.387	122.30 ± 84.18	111.47 ± 76.82	128.12 ± 101.59	0.060
SBP	129.59 ± 18.92	130.31 ± 20.16	129.42 ± 17.39	0.868	125.63 ± 21.98	126.56 ± 23.99	126.22 ± 24.88	0.883
DBP	86.64 ± 13.22	86.79 ± 14.26	86.93 ± 11.98	0.983	81.80 ± 13.81	81.66 ± 14.97	82.17 ± 13.51	0.931
**AGT**	**MM (n = 156)**	**MT (n = 310)**	**TT (n = 252)**	**P**	**MM (n = 142)**	**MT (n = 399)**	**TT (n = 317)**	**P**
Age	46.21 ± 11.44	44.38 ± 10.72	44.24 ± 10.95	0.163	44.89 ± 10.34	45.30 ± 10.95	44.17 ± 10.75	0.368
Weight	73.67 ± 15.35	74.39 ± 13.48	73.87 ± 14.75	0.853	65.35 ± 16.35	64.67 ± 14.78	65.47 ± 15.35	0.773
Height	169.33 ± 7.97	169.72 ± 7.05	169.01 ± 6.90	0.506	158.65 ± 6.42	156.83 ± 6.09^#^	156.25 ± 6.23^#^	0.001
BMI	25.77 ± 4.03	25.86 ± 3.90	25.93 ± 4.45	0.929	26.35 ± 5.49	26.40 ± 5.36	26.97 ± 5.63	0.337
WC	88.94 ± 12.94	88.88 ± 12.05	88.39 ± 13.71	0.878	82.83 ± 17.31	82.91 ± 13.75	83.25 ± 14.44	0.942
WHR	0.92 ± 0.07	0.92 ± 0.07	0.92 ± 0.09	0.735	0.83 ± 0.09	0.84 ± 0.08	0.83 ± 0.08	0.711
Glucose	107.06 ± 32.44	105.54 ± 29.34	105.13 ± 25.13	0.797	98.41 ± 9.96	106.98 ± 40.00^#^	103.70 ± 31.93	0.039
Uric acid	5.40 ± 1.37	5.45 ± 1.44	5.59 ± 1.54	0.345	4.11 ± 1.11	4.23 ± 1.26	4.25 ± 1.38	0.510
TC	215.86 ± 46.06	121.05 ± 55.14	215.53 ± 49.41	0.646	214.16 ± 43.81	217.51 ± 46.00	211.19 ± 43.34	0.157
LDL-c	142.79 ± 41.48	137.75 ± 42.18	141.96 ± 39.12	0.350	142.06 ± 39.46	143.73 ± 41.54	140.40 ± 40.71	0.533
HDL-c	42.28 ± 11.57	40.29 ± 9.85	44.33 ± 14.69*	0.001	48.85 ± 11.60	47.72 ± 11.76	47.95 ± 12.02	0.623
TG	156.52 ± 122.51	175.01 ± 213.28	145.69 ± 99.69	0.098	117.42 ± 75.55	124.00 ± 88.71	109.73 ± 81.12	0.078
SBP	128.08 ± 18.60	128.30 ± 18.89	133.23 ± 20.39^#^*	0.004	120.37 ± 19.54	127.55 ± 23.65^#^	127.27 ± 24.76^#^	0.005
DBP	86.44 ± 14.26	85.57 ± 12.92	88.64 ± 14.31*	0.029	79.24 ± 12.65	82.19 ± 15.25	82.54 ± 13.95	0.061

Age (years); weight (kg); Height (cm); BMI (kg/cm^2^); WC (cm); glucose (mg/dL); uricacid (mg/dL); cholesterol (mg/dL); LDL-c (mg/dL); HDL-c (mg/dL); triglycerides (mg/dL); SBP (mmHg); DBP (mmHg). BMI: body mass index; WC: waist circumference; WHR: waist-to-hip ratio; TC: total cholesterol; LDL-c: low-density lipoprotein cholesterol; HDL-c: high-density lipoprotein cholesterol; TG: triglycerides; SBP: systolic blood pressure; DBP: diastolic blood pressure; ACE: Angiotensin-converting enzyme; AGT: angiotensinogen. ACE: * vs. DD; AGT: * vs. MT; # vs. MM.

Table 2 also details the study population characteristics stratified by sex and AGT polymorphisms (MM, MT and TT genotypes). Neither BMI nor WC changed significantly among genotypes, regardless of sex. Specifically for men, increased HDL-c levels and SBP were observed in the TT genotype compared to MT. In addition, increased DBP was observed for the same genotype (TT) compared to MM and MT. For women, on the other hand, participants with MM genotypes had lower DBP compared to MT and TT genotypes. Women with MT genotypes showed increased glucose levels compared to MM and TT. The same results were found when BMI was replaced by WC in association with ACE and AGT polymorphisms. The remaining characteristics were similar among the stratified groups.

**Figure 1:**
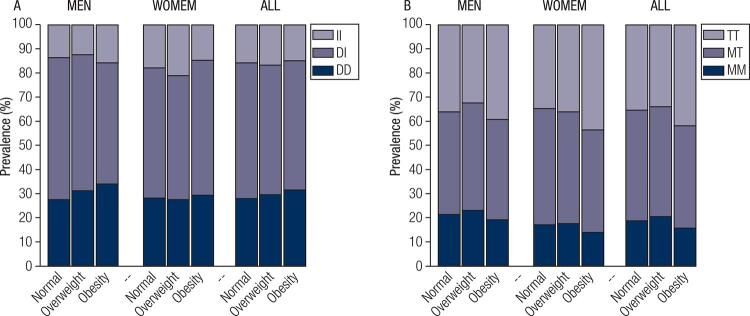
RAS polymorphisms prevalence stratified by body mass index (BMI) and sex (men and women). A) ACE polymorphisms (II, DI or DD). B) AGT polymorphisms (TT, MT or MM). Prevalence is shown as percentage (%).

**Figure 2:**
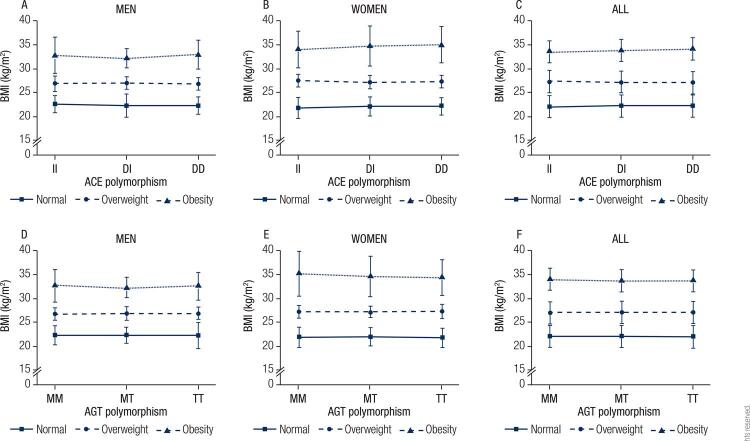
Body mass index values for the different RAS polymorphisms for all sample and according to sex. Age-adjusted BMI distribution according to ACE polymorphism for men (A) and for women (B). Age and sex-adjusted BMI distribution according to ACE polymorphism for all sample (C). Age-adjusted BMI distribution according to AGT polymorphism for men (D) and for women (E). Age and sex-adjusted BMI distribution according to AGT polymorphism for all sample (F).

**Figure 3:**
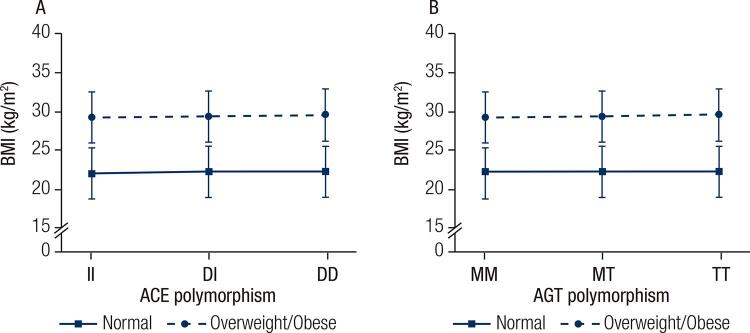
Body mass index values for the different RAS polymorphisms for all sample. Age and sex-adjusted BMI distribution according to ACE polymorphism for men (A) and for women (B).

The frequency of ACE (Figure 1A) and AGT (Figure 1B) genotypes in the study population was stratified by BMI categories (normal, overweight and obese), shown for all participants and divided by sex. The most prevalent ACE and AGT genotypes were DI and MT, respectively, while the least prevalent were II and MM, respectively. However, the prevalence of ACE and AGT genotypes did not vary according to BMI categories, regardless of sex. When the study population was stratified by ACE or AGT (Figures 2A and 2C: men, Figures 2B and 2D: women) genotypes, the adjusted (sex and age) BMI means did not change significantly, regardless of BMI category. The statistical power for these analyses ranged from 0.64 to 0.98 depending on the sample size for each comparison. Similar results were found when analyses were performed based on two BMI categories (normal and overweight/obese), and the study power ranged from 0.77 to 0.99 depending on the comparison (Figure 3).

Although the individual polymorphisms did not correlate significantly with BMI, we hypothesized that the combination of ACE and AGT genotypes could have a significant effect on BMI. Thus, we next analyzed the association between BMI mean and various combinations of ACE and AGT genotypes, even though the power of the study was reduced by the combination of polymorphisms (ranging from 0.41 to 0.91). Table 3 displays the age and sex-adjusted BMI average for each combination of ACE and AGT genotypes. The most prevalent combination was DI/MT, and the least prevalent was II/MM. We did not find significant differences in the sex- and age-adjusted BMI values (*P* value for intragroup comparison = 0.546), regardless of BMI category (*P* value for interaction BMI category vs*.* combined polymorphism = 0.811).

**Table 3:** Age and sex-adjusted BMI values based on combined ACE and AGT genotypes

	NORMAL		OVERWEIGHT/OBESE		*P* for between group comparison	ALL	
	
	Sample size	Mean ± SD	Sample size	Mean ± SD		Sample size	Mean ± SD
II + TT	40	22.1 ± 2.0	49	29.8 ± 3.6	< 0.001	89	26.4 ± 4.9
DI + TT	129	22.1 ± 2.8	184	29.7 ± 4.1	< 0.001	313	26.6 ± 5.2
DD + TT	69	22.2 ± 1.8	87	30.0 ± 4.4	< 0.001	156	26.5 ± 5.2
II + MT	55	22.0 ± 2.1	65	29.0 ± 3.5	< 0.001	120	25.8 ± 4.5
DI + MT	178	22.2 ± 1.8	200	29.4 ± 4.0	< 0.001	378	26.0 ± 4.8
DD + MT	82	22.5 ± 1.7	121	29.7 ± 4.1	< 0.001	203	26.8 ± 4.9
II + MM	15	22.1 ± 2.3	27	29.5 ± 4.8	< 0.001	42	26.8 ± 5.4
DI + MM	76	22.5 ± 1.9	83	29.2 ± 3.9	< 0.001	159	26.0 ± 4.5
DD + MM	40	21.8 ± 2.1	54	29.0 ± 4.0	< 0.001	94	25.8 ± 4.9
*P* for intergroup comparison	0.649		0.283			0.546	
*P* for interaction (BMI category vs. combined polymorphism)					*P* = 0.811		

## DISCUSSION

RAS polymorphisms have been associated with several disorders, especially hypertension, in various study populations (24,25). AGT and ACE polymorphisms have also been investigated regarding their association with obesity (24,26); however, conclusions are still undetermined due to conflicting results. From this perspective, studies of various populations are essential, as genetic heterogeneity would greatly affect these associations. Therefore, we performed a population-based study to verify the association between AGT and ACE polymorphisms with overweight/obesity in a general sample of Brazilian adults. In the present study, we did not find associations between ACE or AGT polymorphisms and overweight/obesity as evaluated by BMI categories (and WC), which are widely used indicators of body fat, especially central adiposity (27).

ACE is an enzyme responsible for cleaving the inactive peptide AngI into the most active peptide of the RAS, AngII. Although the ACE D/I polymorphism is considered nonfunctional by some authors, its relationship with several pathophysiological conditions such as hypertension (28), atherosclerosis (29) and coronary heart disease (30) is extensively discussed in the literature (31). In this sense, the study of the ACE influence on obesity, an important risk factor for cardiovascular diseases, is also relevant.

A study performed by Pan and cols. also evaluated the ACE polymorphism’s association with overweight/obesity and found no close relationship (15), although they reported that BMI was associated with hyperglycemia, hypertension and dyslipidemia in type-2 diabetes patients (15). Motawi and cols. published similar findings in a study of Egyptian women in which ACE polymorphism was not associated with obesity (16). Suchanek and cols. investigated the ACE I/D allele frequency in an obese Czech female population. The authors reported no differences at baseline; however, after a 9-month dietary and physical activity intervention program, II genotyped women had increased metabolism basal rates compared to DD, thus indicating that the I allele may influence individuals’ responses to lifestyle changes (17).

Although some studies have reported no association between ACE polymorphism and obesity, others have found significant associations. For instance, Akin and cols. investigated the frequency of the ACE I/D allele among obese individuals with insulin resistance (IR) and reported that the DD genotype was significantly higher in IR obese individuals than those without IR (32). Similarly, Javaid and cols. reported an increased DD genotype frequency in obese individuals compared to overweight and eutrophic Pakistani individuals. The authors argued that the DD genotype correlates with increased ACE expression, increased AngII levels and, consequently, augmented adipogenesis (33). These findings were corroborated by Mohsen and cols., who reported an increased DD genotype frequency in overweight and obese Saudi individuals. They also attributed the increased DD frequency to the possible modulation of fat metabolic pathways, which may increase individuals’ susceptibility to overweight/obesity (34).

We have also investigated AGT polymorphism’s influence on overweight and obesity. In our study, no close relationship between AGT genotypes and BMI and WC was observed, corroborating previous findings (24). However, a study performed by Prat-Larquemin and cols. reported that although the AGT genotypes were not associated with fat mass, the T allele was associated with increased adipocyte size, evidencing increased adipocyte capacity to store fat, a hallmark characteristic of obesity (26). On the other hand, a heritage family study performed by Rankinen and cols. reported that the T allele was associated with increased body fat mass in women and that fat mass may influence this polymorphism’s already well-described effects on blood pressure (35).

Researchers have already investigated the AGT expression profile related to obesity; Umemura and cols. reported a correlation between increased plasma AGT levels with BMI and blood pressure in obese individuals (36). Similarly, Giacchetti and cols. investigated AGT mRNA expression in adipose tissue of obese individuals and reported a significant association between AGT expression and BMI in visceral adipose tissue (37). However, as previously reported, data on AGT polymorphism’s influence on obesity are scarce in the literature, while this gene polymorphism’s effects on blood pressure are well established (38). The investigation of the AGT mutation’s influence on overweight/obesity deserves attention, as the RAS polymorphism’s detection could help to track patients at increased risk of developing obesity-associated conditions and allow the implementation of preventive measures to avoid unwanted outcomes.

The present study may differ from those reported in the literature due to its population-based characteristic (n = 1,567), which is important for result generalization. The Brazilian population is highly admixed, and our sample included participants of European and African descent. The most prevalent group, however, was composed of admixed subjects, generally referred to as “brown.” However, some limitations should be pointed out. Firstly, the small sample size for the ACE and AGT combined analysis could have hindered positive associations, even though the means were fairly similar among groups and the power for that analysis was acceptable. Thus, a study with a large sample size is required to draw a reliable conclusion. Secondly, the cross-sectional design of our study does not allow us to establish causal relationships between the studied variables and the ACE and AGT polymorphic allele frequencies.

In conclusion, we observed no relationship between the ACE I/D and AGT M/T polymorphisms and overweight/obesity in a population-based study accessed via BMI (and WC) categories. Additional studies with larger populations should be performed to elucidate this combined relationship, as it is still controversial in the literature.
